# Individual-Area Relationship Best Explains Goose Species Density in Wetlands

**DOI:** 10.1371/journal.pone.0124972

**Published:** 2015-05-21

**Authors:** Yong Zhang, Qiang Jia, Herbert H. T. Prins, Lei Cao, Willem Fred de Boer

**Affiliations:** 1 Resource Ecology Group, Wageningen University, Wageningen, The Netherlands; 2 School of Life Science, University of Science and Technology of China, Hefei, Anhui, China; 3 State Key Laboratory of Urban and Regional Ecology, Research Center for Eco-Environmental Science, Chinese Academic of Sciences, Beijing, China; University of Sydney, AUSTRALIA

## Abstract

Explaining and predicting animal distributions is one of the fundamental objectives in ecology and conservation biology. Animal habitat selection can be regulated by top-down and bottom-up processes, and is mediated by species interactions. Species varying in body size respond differently to top-down and bottom-up determinants, and hence understanding these allometric responses to those determinants is important for conservation. In this study, using two differently sized goose species wintering in the Yangtze floodplain, we tested the predictions derived from three different hypotheses (individual-area relationship, food resource and disturbance hypothesis) to explain the spatial and temporal variation in densities of two goose species. Using Generalized Linear Mixed Models with a Markov Chain Monte Carlo technique, we demonstrated that goose density was positive correlated with patch area size, suggesting that the individual area-relationship best predicts differences in goose densities. Moreover, the other predictions, related to food availability and disturbance, were not significant. Buffalo grazing probably facilitated greater white-fronted geese, as the number of buffalos was positively correlated to the density of this species. We concluded that patch area size is the most important factor determining the density of goose species in our study area. Patch area size is directly determined by water levels in the Yangtze floodplain, and hence modifying the hydrological regimes can enlarge the capacity of these wetlands for migratory birds.

## Introduction

Explaining and predicting animal distributions is one of the fundamental objectives in ecology and conservation biology. Despite intensive research efforts during the past decades, this issue remains incompletely understood, partly because population density of animals can be determined by a variety of abiotic and biotic determinants that interact and operate at different spatial scales. Particularly, biotic interactions, such as top-down (i.e., predation) and bottom-up (i.e., food availability and quality) factors, operate across trophic levels [1] under influence of competitive and facilitative interactions [2–4]. The effects of those determinants may vary among species through allometric responses [5]. Predictions derived from allometric relationships state that animals differing in body size respond differently to top-down and bottom-up factors based on e.g., physiologic and digestive consequences of body size. Therefore, understanding the different responses among species to those determinants is crucial for conservation. In this paper, using two herbivorous goose species, we aim to answer two questions: if and how top-down and bottom-up factors affect goose density and if the effects vary between species, offering insight into the underlying factors that conservation strategies should cover.

Eastern China supports more than two million migratory waterbirds during the non-breeding seasons, of which more than one million overwinter in the Yangtze River floodplain [6]. Anatidae species such as geese mainly feed on recessional grassland in the Yangtze floodplain in winter. A primary factor determining goose population density would be the extent of available habitat. The sizes of the patches of available habitat change with the water level fluctuations and thereby affect the habitat selection of these birds, which can be described by an individual-area relationship (IAR). The IRA describes the relationship between animal population size and area [7]. Positive IRAs are often found [7, 8], which is in line with the resource concentration hypothesis [9]. The resource concentration hypothesis, introduced by researchers on herbivorous insects, states that larger areas of host plants should attract more herbivores [9]. Movements of consumers between patches are also used to explain IARs [7, 10], as animals can move to larger or richer patches if this is beneficial in terms of their own net foraging success, and thereby affect the availability of resources in patches, often resulting in a positive IAR, consistent with the ideal free distribution [11]. Hence, the capacity of the Yangtze floodplains to accommodate migratory birds might be negatively affected if the availability or the size of these recessional grasslands is reduced.

Forage quantity and structural heterogeneity play an important role in determining the animal’s patch selection as animals select patches offering the highest forage intake such as predicted by the optimal foraging theory [12–17]. Goose species generally display a Type IV functional response, which is a dome-shaped curve with a maximum intake rate at intermediate forage biomass, and a decreasing intake at higher biomass densities. Smaller species tend to select lower biomass areas as their maximum intake is reached earlier than for larger species [12, 18]. In addition, habitat heterogeneity, such as horizontal variation in available forage biomass, tend to increase species richness [19, 20], but the effect of habitat heterogeneity differs among species [21]. Habitat heterogeneity can also negatively affect the forage efficiency of grazers by increasing searching and handling times [22, 23]. Herbivores, such as many overwintering waterbird species (e.g., *Anser* spp., *Anas* spp.), generally have a lower intake rate and consequently reach lower population size while feeding on heterogeneous swards compared to homogenous swards [12, 24, 25].

These ecological factors are important in determining animal distribution and density. In addition, anthropogenic activities (e.g., agriculture, aquaculture and livestock breeding) are playing an increasing role [26–29]. Such activities are found to be strongly correlated with habitat selection of geese, and can have both negative or positive effects on geese densities [17, 30–33].

Species normally react differently to ecological and anthropogenic factors, and this reaction is often mediated by differences in body size as indicated by allometric scaling laws [34, 35]. The effect of forage quantity and structural heterogeneity is influenced by body size, as smaller sized species generally select areas with a lower forage quantity but with more homogenous resources [12]. Larger species are more sensitive to human disturbances [26, 36]. However, species can also react positively to human factors, for instance, the grazing by domestic larger grazers such as cattle or buffalo, can facilitate resource availability for smaller grazer species by changing resource structure and nutrient content [37–40]. For instance, it has been reported that the density of geese was higher in areas with a higher sheep density [41].

In this paper, we analysed the effects of anthropogenic and ecological determinants on goose species density in wetland in China, using two migratory grazing goose species, namely bean goose (*Anser fabalis*, body weight: 3100 g) and greater white-fronted goose (*Anser albifrons*, body weight: 2400 g), which both rely on the same food resource and habitat in the same period. We tested several hypotheses:

- the individual-area relationship hypothesis: we predicted that goose density increases with an increasing area of the exposed land;
- the food resource hypothesis: we predicted that goose density increases with increasing forage quantity until a certain threshold. Habitat heterogeneity is expected to negatively affect goose density, and the smaller species would be more sensitive to such heterogeneity than the larger one;
- the disturbance hypothesis: we predicted a negative effect of human disturbance (i.e., the presence of domestic geese and boats) on wild goose density for both species, but with a stronger reaction for the larger species, and a positive, facilitative effect by the number of water buffalo.

## Materials and Methods

### Study area

Shengjin Lake National Nature Reserve (30°16′–30°25′N, 116°59′–117°12′E), located on the southern bank of the Yangtze River, is an important wetland in the Yangtze floodplain for wintering waterfowl. In summer, the maximum lake area is about 14,000 ha, in winter, as the water levels decline, the lake area decreases to about 3,400 ha. Water comes from three smaller rivers flowing directly into the lake and from the Yangtze River via the Huangpen Sluice built in 1965 (Cheng and Xu, 2005). The sluice was built to regulate the water level for facilitating agricultural activities and to control floods. The average annual rainfall is about 1600 mm, with most rain falling from March to August, and the average annual temperature is 16.1°C, with an average January temperature of 4.0°C.

### Ethics statement

Field permit of this research was granted by the Shengjin Lake National Reserve, Anhui Province, China. This study was approved by the Animal ethics committee University of Science and Technology of China (Approval number: USTCACUC1205052).

### Survey methods

**Fig 1:**
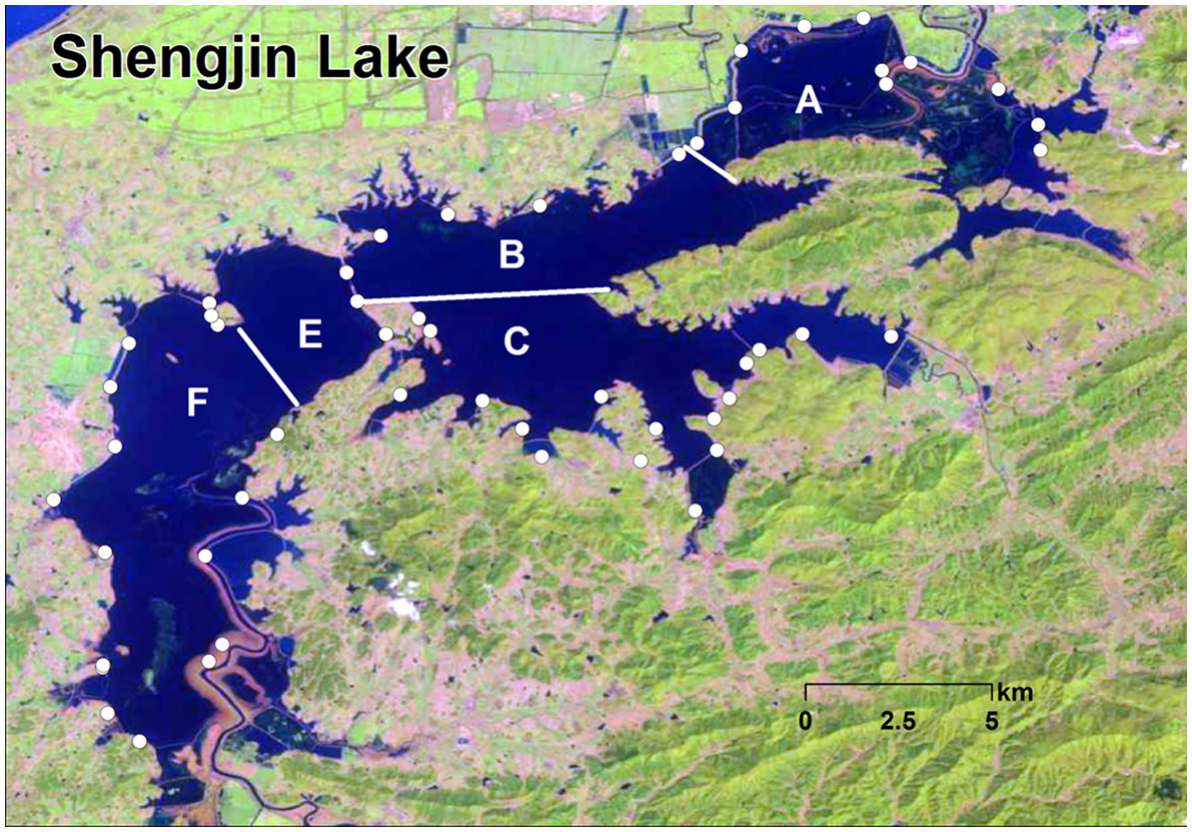
Map of Shengjin Lake and the five discrete survey areas. The white circles indicate the 56 counting points and the white lines indicate the counting area boundary (Source:http://eros.usgs.gov/#).

Shengjin Lake was divided into five discrete survey areas (Fig 1). The major factors considered in deciding the size of a survey area was that it had clear boundaries, defined by natural and artificial features and the entire lake could be adequately surveyed by two teams of 2 persons in two days. Within each survey area, discrete sub-areas were identified by natural boundaries and features which enabled sub-areas to be completely surveyed. Each sub-area could be surveyed from a fixed counting location (Fig 1). The lake was surveyed every 16 days from 2008 to 2013 in winter, depending on the satellite passing and on local weather conditions. Survey areas A and B were surveyed on the same day by one group of observers, while area C was surveyed at the same time by another group. Areas E and F were surveyed by the two groups of observers simultaneously, one surveying the west side and the other the east side of the lake. To avoid double counting, cell phone communication was used during the survey. In case of poor weather conditions, with e.g., fog or rain, an additional day was needed to complete the survey (7 out of 22 surveys). The “look-see” counting method is commonly used to count waterbirds [42] and was therefore used in this study. For each sub-area, the number of bean goose and number of greater white-fronted goose was recorded. In case of geese moving within the sub-area, we waited until all geese had settled and no geese were flying around anymore. For both species, we recorded the sizes of the different sub-flocks as this method can reduce the error when counting large numbers of birds [43]. Time spent on each counting point was different, from 5–20 min, except four counting sub-area F where around 60 min was needed to count all birds. In addition, potential disturbance factors (Table 1) were also recorded for each sub-area. After each survey, a distribution map was drawn with reference to species, bird numbers, location and date and time. A detailed description of the survey methodology can be found in Cao et al [44].

**Table 1:** The potential predictor variables and their abbreviations used to analyse differences in goose densities. H_0_ indicates expected relationship. +: positive; -: negative. NDVI: Normalized Difference Vegetation Index.

Hypothesis	Variables	Unit	Explanation	Range	Abbreviations	H_0_
Individual-area hypothesis	Patch area	km^2^	Calculated from satellite images	0.004 ~ 4.867	PA	+
Food resource hypothesis	Total biomass	g/m^2^	Calculated from NDVI data using built equation	0.000 ~ 122.928	BIO	+
	Square of total biomass	g/m^2^			BIO^2^	-
	Coefficient of variation	no	Calculated by standard deviation of NDVI divided by mean NDVI	0.000 ~ 1.011	CV	-
Disturbance hypothesis	Number of buffaloes	no		0 ~ 461	BUFF	+
	Number of boats at anchor	no		0 ~ 55	BA	-
	Number of domestic goose	no		0 ~ 4000	GOOSE	-

### Satellite image processing

Normalized Difference Vegetation Index (NDVI) was calculated to represent forage quantity using Multispectral HJ-1A, Landsat Thematic Mapper (TM) and Enhanced Thematic Mapper + (ETM+) images (with a consistent spatial resolution of 30 m). We selected the images (with less than 10% cloud cover) that were recorded around our survey dates (S1 and S2 Tables). Due to the sensor failure of Landsat 7 in 2003, parts of the data were lost on the edges of the ETM+ image (USGS, 2013). Fortunately, such data loss only accounted for about 5% of our study area, and a gap-filling method based on local linear histogram matching (Scaramuzza 2004) was used to fix the missing data.

After fixing the missing ETM+ data, we conducted image calibration (converting digital numbers to radiance) and atmospheric correction (using a Fast Line-of-sight Atmospheric Analysis of Hypercubes, FLAASH) [45]. Geometric correction was applied using second-order polynomials with an accuracy of less than 0.5 pixels Square Mean Root Error (SMRE). Pseudo Invariant Features (PIF) was used to normalize all images to allow for comparison between datasets [46].

We adopted the Supported Vector Machines (SVMs) method to discriminate between water and land within our study area because of their proven efficiency and accuracy in binary classification [47]. Further, we applied a NDVI threshold to distinguish between bare soil and meadows. To determine this threshold value, NDVI values were plotted against log-transformed vegetation biomass, which was measured in each winter month during 2010–2012. Eventually we selected 0.18 as the threshold to distinguish between bare soil and meadows (S1 Fig). Image processing was performed in ENVI 4.8 and ArcGIS 10.0 software.

### Statistical analysis

The independent variables that potentially affected geese habitat selection and density, their abbreviations and predicted effects are given in Table 1.

Vegetation biomass of the grassland is a direct indicator of forage quantity. However, field measurement of biomass is not available for each survey date, but we found a strong empirical relationship between measured total biomass (log transformed g/m^2^) and NDVI (including both linear and quadratic term) using regression analysis (see Results). Hence, we calculated vegetation biomass based on NDVI data.

Generalized Linear Mixed Models using Markov Chain Monte Carlo techniques (MCMCglmm) [48] were performed to separately test our predictions. A total of 22 surveys’ data across five wintering periods were used depending on the satellite images quality and the passing data (S2 Table). Because both goose species wintered in our research area from late September to the end of March, we only used the survey data from within this wintering period. The number of birds on the water was excluded from the counts, as we intended to measure the effect of the size of the patch area. We ran the analysis using zero-inflated models (family = zipoisson) and nonzero-inflated models (family = poisson) for both species separately and compared the model fit using the deviance information criterion (DIC). The model with lowest DIC was considered as the appropriate model [49]. We performed 30,000 iterations as burn-in, followed by 300,000 runs with a thinning interval of 100. The density of bean goose and greater white-fronted goose were dependent variables. The independent variables are listed in Table 1. Survey time (year and month) and site (counting points) were random factors. We checked for autocorrelation between samples, but found that autocorrelation was of little influence (all positive values were close to zero). We also tested for spatial autocorrelation of the residuals using Moran’s I index, and found little evidence for spatial autocorrelation (S3 Table). Correlation between pairs of independent variables was weak (all pairwise correlations, |r| < 0.01), indicating that there was no multicollinearity problem. Statistical analyses were conducted in R 2.13.0 with the package MCMCglmm.

## Results

A strong positive relationship was found between total biomass (g/m^2^) and NDVI as: log(g/m^2^+1) = 15.57 * NDVI-9.77 * NDVI^2^–1.26 (R^2^_adj_ = 0.56, F_2,232_ = 152.1, P < 0.001), which indicated that NDVI was a good proxy of forage biomass.

The nonzero-inflated model fitted our data best for both species according to the DIC value (Table 2) and was therefore used in further analysis. The predictions derived from the individual area relationship hypothesis were confirmed as patch area size (PA) had a significantly positive effect on the geese density for both species (Tables 3 and 4), whereas food and disturbance variables were not significant. Also the number of buffalos had a positive effect on the density of greater white-fronted goose (Table 4).

**Table 2:** Comparison of the values for the deviance information criterion (DIC) between models (MCMCglmm) that were built using a zero-inflated (zipoisson) and nonzero-inflated (poisson) distribution.

Hypothesis	DIC value			
	Bean goose		Greater white-fronted goose	
	zero-inflated	nonzero-inflated	zero-inflated	nonzero-inflated
Individual-area relationship hypothesis	3796.6	3369.3	1858.8	1428.5
Food resource hypothesis	4215.5	3365.6	1874.4	1428.6
Disturbance hypothesis	4184.1	3368.3	1862.6	1426.3

**Table 3:** Summary of the effects of dependent variables on bean goose density as generated by the MCMCglmm model for each of the hypotheses and independent variables, with coefficients and p-values.

Hypothesis	Variables	Coefficient	Lower 95% CI	Upper 95% CI	P-value
Individual-area hypothesis	PA	0.962	0.106	1.866	0.036
Food resource hypothesis	BIO	-0.034	-0.108	0.045	0.373
	BIO^2^	0.000	-0.001	0.001	0.496
	CV	-1.828	-8.550	3.974	0.568
Disturbance hypothesis	BA	0.066	-0.081	0.210	0.356
	GOOSE	-0.000	-0.003	0.002	0.801
	BUFF	0.012	-0.002	0.025	0.077

CI = confidence interval of the coefficient. For abbreviation of dependent variables see Table 1.

**Table 4:** Summary of the effects of dependent variables on greater white-fronted goose density as generated by the MCMCglmm model for each of the hypotheses and independent variables, with coefficients and p-values.

Hypothesis	Variables	Coefficient	Lower 95% CI	Upper 95% CI	P-value
Individual-area hypothesis	PA	1.739	0.669	2.939	0.003
Food resource hypothesis	BIO	-0.005	-0.116	0.096	0.930
	BIO^2^	-0.000	-0.001	0.001	0.704
	CV	-0.358	-10.140	8.890	0.936
Disturbance hypothesis	BA	-0.169	-0.531	0.170	0.302
	GOOSE	-0.000	-0.003	0.002	0.768
	BUFF	0.020	0.005	0.035	0.005

CI = confidence interval of the coefficient. For abbreviation of dependent variables see Table 1.

## Discussion

In this study, we tested the predictions derived from three hypotheses in explaining the variation in densities of two grazing goose species in Yangtze wetlands. We demonstrated that the patch area size (PA) was positively correlated with the density of both goose species, indicated that our data strongly supported the individual-area relationship. For both species, we failed to detect any support for the food resource and disturbance hypothesis, as all food and disturbance variables were not significant, although the number of domesticated buffalos had a positive effect on the density of greater white-fronted goose. Our results showed that patch area size is the most important factor in explaining spatial differences in bird distribution and densities, supporting the individual-area hypothesis, which is in line with the findings of Connor and co-workers [7]. Connor et al. [7] discussed that their analyses may be biased. We conducted our bird censuses in a systemic way, using point counts. The counting points were selected carefully to cover a certain sub-area, determined by natural and artificial boundaries. Both our study species are larger herbivores which are easily detectable. The resource concentration hypothesis, first introduced for herbivorous insects [9], is often employed to explain IARs. The hypothesis states that larger areas of host plants should attract more herbivores, because the animals are more likely to find the plants and stay longer. An alternative explanation for this relationship is that predation risk is higher in smaller patches than in larger ones [9, 50].

Our study did not find any support for the food resource hypothesis; biomass was even negatively, but not significantly, correlated with the bird densities of both species (Tables 3 and 4). These results are not consistent with the ideal free distribution which predicts that consumer density is positively related to resource availability. This may be explained by differences in forage quality and the animal’s digestion system. Plant quality generally decreases over the growing season with increasing biomass [51]. Grazing wildfowl are sensitive to variation in forage quantity and quality [52]. Goose species have a poor digestion system and may not be able to tolerate low forage quality, Vegetation heterogeneity (CV) was also negatively correlated with bean goose and greater white-fronted goose density, but this was also not significant (Tables 3 and 4). Foraging on more homogeneous area can reduce searching time [23] and hence offer a higher peck rate to satisfy the relatively high daily energy demands of these goose species. However, the weak negative effect indicated that vegetation heterogeneity is not the main factor that determines goose species density in these wetlands.

We also failed to detect a significant effect of disturbance related factors. As Shengjin lake is a national nature reserve in China and also one of the Ramsar wetlands, it is also relatively better managed. However, we still found negative slopes for the effects of domestic goose (GOOSE) and boats at anchor (BA) for both species, suggesting that these factors might play a weaker role in determining goose densities. Water buffalo (BUFF), the dominant livestock species in the study area, also forage on these grasslands. A positive correlation between number of buffalos and goose density was found for both species, suggested that goose density increased with number of water buffalo especially for greater white-fronted goose, indicating that water buffalos can facilitate geese. A Type IV functional response for these two goose species was found in previous studies [12, 53], which suggests that their density is expected to decrease once a certain optimal level of forage biomass has been surpassed. Grazing livestock such as buffalos may reduce forage biomass, change the vegetation structure and increase the availability of nutritious regrowth, and thereafter facilitate goose species grazing [39, 54]. Moreover, foraging with buffalos may also lead to an earlier detection of predators, such as dogs.

Our study generated strong support for the individual-area relationship, and temporal and spatial differences in resource availability or disturbance seem to play no role in determining the differences in goose density. This result highlights the importance of patch area size in determining the animal’s habitat choice. To safeguard China’s wetlands biological diversity, conservation biologists and policymakers often face a dilemma in prioritizing conservations actions, as habitat selection of wetlands birds is complex, assumed to be regulated by ecological and anthropogenic factors. Our results indicate that simply increasing patch area size is an eminent management action, as one larger exposed grassland area is more attractive for migratory goose than several smaller areas with the same total area. The exposure of recessional grasslands is directly determined by fluctuations of water level. Hence, in order to enlarge the capacity of the Yangtze wetlands and better protect wintering wildfowl, hydrological regimes could be optimized. Moreover, our results support the knowledge that habitat fragmentation may negatively affect animal densities. Hence, we suggest that water level management schemes should be optimized to both address the factors that determine the wetland suitability for migratory birds, such as through a reduction in habitat fragmentation and an increase in the area of recessional grasslands, while also addressing the need for water for irrigation and aquacultural purposes and flood protection.

## Supporting Information

S1 Fig Scatterplot of vegetation total biomass (g/m2) and NDVI. Vegetation total biomass was ln-transformed.
(DOCX)

S1 Table Date of acquired satellite images and total biomass data used in the analysis for predicting forage total biomass from differences in NDVI. (DOCX)

S2 Table Image date and information of acquired satellite images and their corresponding survey date. (DOCX)

S3 Table Moran’s I values of residuals for the test of spatial autocorrelation in the final model both for each survey. BG = bean goose; GWFG = greater white-fronted goose. * P< 0.05; ** p < 0.01; *** p < 0.001.
(DOCX)
