# Supplementary material for: Individual-Area Relationship Best Explains Goose Species Density in Wetlands
Source: PLoS One. 2015 May 21;10(5):e0124972. doi: 10.1371/journal.pone.0124972 (PMC4440642; doi:10.1371/journal.pone.0124972)

**S1 Figure.** Scatterplot of vegetation total biomass (g/m^2^) and NDVI. Vegetation total biomass was ln-transformed.


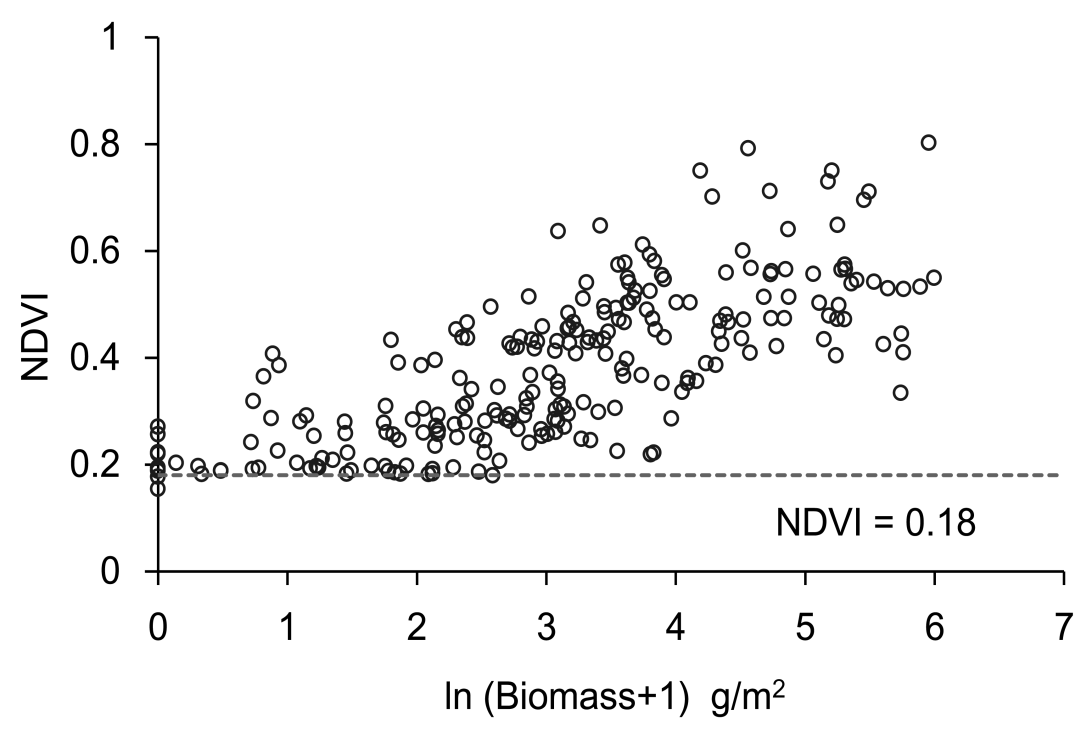

Supplement: S1 Fig — Vegetation total biomass was ln-transformed. (DOCX) [file pone.0124972.s001.docx]
