# Supplementary material for: Individual-Area Relationship Best Explains Goose Species Density in Wetlands
Source: PLoS One. 2015 May 21;10(5):e0124972. doi: 10.1371/journal.pone.0124972 (PMC4440642; doi:10.1371/journal.pone.0124972)
Supplement: S1 Table — (DOCX) [file pone.0124972.s002.docx]

**S1 Table.** Date of acquired satellite images and total biomass data used in the analysis for predicting forage total biomass from differences in NDVI.

| Year | HJ1A CCD2 | HJ1B CCD1 | HJ1B CCD2 | Landsat ETM+ |
| --- | --- | --- | --- | --- |
| 2010 | Oct, Nov | Dec |  |  |
| 2011 |  |  | Nov | Dec |
| 2012 | Feb |  |  |  |
