# Supplementary material for: Individual-Area Relationship Best Explains Goose Species Density in Wetlands
Source: PLoS One. 2015 May 21;10(5):e0124972. doi: 10.1371/journal.pone.0124972 (PMC4440642; doi:10.1371/journal.pone.0124972)
Supplement: S2 Table — (DOCX) [file pone.0124972.s003.docx]

**S2 Table.** Image date and information of acquired satellite images and their corresponding survey date

| Images date | Satellite | Sensor | Survey date |
| --- | --- | --- | --- |
| 02-Dec-08 | Landsat | ETM+ | 06-Dec-08 |
| 18-Dec-08 | Landsat | ETM+ | 15-Dec-08 |
| 31-Oct-09 | HJ 1 | A CCD2 | 31-Oct-09 |
| 05-Dec-09 | Landsat | ETM+ | 04-Dec-09 |
| 21-Dec-09 | HJ 1 | A CCD1 | 18-Dec-09 |
| 14-Jan-10 | Landsat | TM | 04-Jan-10 |
| 23-Feb-10 | Landsat | ETM+ | 16-Feb-10 |
| 19-Mar-10 | Landsat | TM | 16-Mar-10 |
| 31-Oct-10 | HJ 1 | A CCD2 | 21-Oct-10 |
| 06-Nov-10 | HJ 1 | A CCD1 | 05-Nov-10 |
| 22-Nov-10 | Landsat | ETM+ | 22-Nov-10 |
| 08-Dec-10 | Landsat | ETM+ | 08-Dec-10 |
| 21-Dec-10 | HJ 1 | B CCD1 | 23-Dec-10 |
| 25-Jan-11 | Landsat | ETM+ | 24-Jan-11 |
| 21-Feb-11 | HJ 1 | B CCD2 | 25-Feb-11 |
| 23-Mar-11 | HJ 1 | A CCD1 | 14-Mar-11 |
| 19-Oct-11 | HJ 1 | B CCD2 | 16-Oct-11 |
| 12-Nov-11 | HJ 1 | B CCD2 | 09-Nov-11 |
| 25-Nov-11 | Landsat | ETM+ | 25-Nov-11 |
| 11-Dec-11 | Landsat | ETM+ | 10-Dec-11 |
| 18-Oct-12 | HJ 1 | B CCD2 | 11-Oct-12 |
| 12-Dec-12 | HJ 1 | A CCD1 | 14-Dec-12 |
