# Supplementary material for: Individual-Area Relationship Best Explains Goose Species Density in Wetlands
Source: PLoS One. 2015 May 21;10(5):e0124972. doi: 10.1371/journal.pone.0124972 (PMC4440642; doi:10.1371/journal.pone.0124972)
Supplement: S3 Table — BG = bean goose; GWFG = greater white-fronted goose. * P< 0.05; ** p < 0.01; *** p < 0.001. (DOCX) [file pone.0124972.s004.docx]

**S3 Table.** Moran’s I values of residuals for the test of spatial autocorrelation in the final model both for each survey. BG = bean goose; GWFG = greater white-fronted goose. * P< 0.05; ** p < 0.01; *** p < 0.001.

|  |  | Moran’s I value for three models | | | | | | | |
| --- | --- | --- | --- | --- | --- | --- | --- | --- | --- |
| Survey date |  | individual-area relationship | |  | Food resource model | |  | Disturbance model | |
|  |  | BG | GWFG |  | BG | GWFG |  | BG | GWFG |
| 06-Dec-08 |  | -0.062 | -0.013 |  | -0.156* | -0.010 |  | 0.028 | 0.015 |
| 15-Dec-08 |  | -0.021 | 0.005 |  | 0.024 | -0.018 |  | 0.015 | 0.078 |
| 31-Oct-09 |  | -0.026 | 0.010 |  | 0.009 | -0.012 |  | -0.015 | -0.059 |
| 04-Dec-09 |  | 0.095* | -0.133* |  | 0.034 | -0.031 |  | 0.008 | -0.133* |
| 18-Dec-09 |  | -0.051 | 0.001 |  | 0.055 | -0.042 |  | -0.086 | 0.180** |
| 04-Jan-10 |  | -0.055 | -0.010 |  | 0.065 | -0.012 |  | 0.081 | 0.016 |
| 16-Feb-10 |  | 0.049 | 0.035 |  | -0.029 | 0.024 |  | 0.042 | 0.065 |
| 16-Mar-10 |  | 0.030 | -0.027 |  | 0.034 | -0.014 |  | -0.014 | -0.004 |
| 21-Oct-10 |  | -0.043 | -0.021 |  | 0.064 | -0.051 |  | 0.060 | 0.002 |
| 05-Nov-10 |  | 0.045 | 0.030 |  | 0.103* | 0.104* |  | 0.048 | 0.053 |
| 22-Nov-10 |  | -0.023 | 0.005 |  | 0.025 | -0.022 |  | 0.087* | 0.112* |
| 08-Dec-10 |  | 0.066 | -0.016 |  | 0.087 | 0.160* |  | 0.062 | -0.012 |
| 23-Dec-10 |  | -0.005 | 0.015 |  | 0.036 | -0.053 |  | 0.086 | 0.005 |
| 24-Jan-11 |  | -0.026 | -0.004 |  | 0.026 | 0.006 |  | 0.009 | -0.081 |
| 25-Feb-11 |  | -0.098 | 0.033 |  | -0.004 | -0.005 |  | -0.055 | 0.086 |
| 14-Mar-11 |  | 0.013 | -0.020 |  | -0.041 | -0.020 |  | 0.046 | -0.048 |
| 16-Oct-11 |  | -0.060 | 0.023 |  | -0.032 | -0.016 |  | 0.095* | -0.000 |
| 09-Nov-11 |  | -0.042 | -0.016 |  | -0.024 | -0.088 |  | 0.003 | 0.030 |
| 25-Nov-11 |  | 0.008 | 0.012 |  | -0.035 | -0.068 |  | -0.043 | -0.033 |
| 10-Dec-11 |  | -0.051 | -0.015 |  | -0.081 | 0.234*** |  | -0.038 | 0.012 |
| 11-Oct-12 |  | -0.101 | -0.013 |  | -0.034 | 0.060 |  | -0.036 | -0.036 |
| 14-Dec-12 |  | 0.027 | -0.033 |  | 0.140* | 0.081 |  | 0.060 | -0.062 |
